# Survival benefit of radiotherapy to patients with small cell esophagus carcinoma - an analysis of Surveillance Epidemiology and End Results (SEER) data

**DOI:** 10.18632/oncotarget.6764

**Published:** 2015-12-26

**Authors:** Yaqi Song, Wanwei Wang, Guangzhou Tao, Weiguo Zhu, Xilei Zhou, Peng Pan

**Affiliations:** ^1^ Department of Radiation Oncology, Huai'an First People's Hospital, Nanjing Medical University, Nanjing, Huai'an 223300, China

**Keywords:** esophageal cancer, small cell carcinoma, radiotherapy, prognostic factors, stage

## Abstract

**Background and Aims:**

Small cell esophageal carcinoma (SCEC) is a rare malignant tumor. So far, few studies are found to research the effect of radiotherapy (RT) to it. This study is designed to explore the prognostic factors, and analyze survival benefit of RT to patients with SCEC.

**Results:**

Patients with SCEC were more likely to be in female, older, higher disease stage than those with non-small cell esophageal carcinoma. RT was used in more than 50% SCEC patients. RT tended be reduced as the disease stage raise in SCEC. Univariate and multivariate analysis showed that age, year, disease stage, and RT were the prognostic factors of survival (*P* < 0.05). RT reduced nearly 75% risks of death in localized stage (*P* < 0.05), nearly 50% risks of death in regional stage (*P* > 0.05) and nearly 30% risks of death in distant stage (*P* > 0.05).

**Methods:**

SCEC patients between 1973 and 2012 were searched from the Surveillance Epidemiology and End Results (SEER) data. Clinical factors including age, year, sex, race, stage, surgery, and RT were summarized. Univariate and multivariate analysis were performed to explore the independent prognostic factors of SCEC. Cox regression survival analysis was performed to evaluate the effect of RT to SCEC based on different stages.

**Conclusions:**

Stage, age, year, and RT are independent prognostic factors of SCEC. Survival benefit of RT exists in any disease stage, but is only statistically significant in localized stage of SCEC.

## INTRODUCTION

Small cell carcinoma (SCC) is an aggressive progression, high incidence of metastasis, poor prognosis malignancy. It commonly occur in lung, and is usually regarded as a systemic disease [1, 2]. Small cell esophageal carcinoma (SCEC), originated from esophageal tissue, is a rare kind of SCC [3]. Hence, it is hard to get enough patients of SCEC for clinical trial. Current therapeutic schedule for SCEC, a combination of systemic therapy and locoregional treatment, is mainly from the treatment experience of small cell lung cancer (SCLC) [1, 4, 5]. Here, Chemotherapy, as a systemic therapy, is very import for the metastatic ability of SCEC [6, 7]. Radiotherapy and surgery both are locoregional therapy. Their efficacy of SCEC are not very clear, and need further studies [7, 8, 9].

The Surveillance, Epidemiology, and End Results (SEER) Program is a professional cancer related database set up by the National Cancer Institute (NCI) in the United States. It collects and reports cancer incidence and survival data from population-based cancer registries and covers approximately 28% of the US population. With large information of cancer, it is an important tool to analyze rare carcinoma.

In view of above, we used SEER data for the analysis of SCEC. Purpose to explore prognosis factors and efficacy of radiotherapy to SCEC.

## RESULTS

A total of 60385 esophagus carcinoma cases were selected from the SEER database, of which, 352 patients (0.58%) were identified as small cell cancer. A detailed listing of the patient characteristics and pathological features was presented in Table 1. From it we found that Compared with non-small cell esophageal cancer (NSCEC), the small cell esophagus cancer (SCEC) was more likely to be distant metastatic (59.12% Vs 38.51%, *P* < 0.001). Patients with SCEC had a fewer median survival (8 Vs 10 months, *P* < 0.001) and a higher proportion of women (40.06% Vs 23.86%, *P* < 0.001) than those with NSCEC. Nearly 50% of patients with SCEC accepted radiation therapy, while only fewer than 10% of them were treated with surgery. So radiotherapy is a most important locoregional treatment method of SCEC.

**Table 1:** Characteristics of SCEC patients from SEER database

Variable		NSCEC	SCEC	χ^2^	*P*-value^ψ^
MS (mo)		10	8	20	< 0.001***
Year	1973–1992	14342 (23.89%)	92 (26.14%)	1.691	0.429
	1993–2002	16572 (27.60%)	101 (28.69%)		
	2003–2012	29119 (48.51%)	159 (45.17%)		
Age	70−	36125 (60.18%)	194 (55.11%)	3.532	0.060
	70+	23908 (39.82%)	158 (44.89%)		
Race	Black	8886 (14.84%)	65 (18.57%)	3.908	0.142
	White	47802 (79.84%)	266 (76.00%)		
	Other	3182 (5.32%)	19 (5.43%)		
Stage	Localized	13899 (27.65%)	63 (21.28%)	54.369	< 0.001***
	Regional	17006 (33.84%)	58 (19.60%)		
	Distant	19355 (38.51%)	175 (59.12%)		
Sex	Male	45712 (76.14%)	211 (59.94%)	49.548	< 0.001***
	Female	14321 (23.86%)	141 (40.06%)		
Surgery	None	41570 (72.02%)	311 (91.47%)	62.672	< 0.001***
	Surgery	16153 (27.98%)	29 (8.53%)		
Radiation	None	25696 (43.76%)	175 (49.86%)	5.017	0.025*
	Radiation	33018 (56.24%)	176 (50.14%)		

Abbreviations: MS = median survival.

ψ MS: log-rank test; others: chi-square test

* *P* < 0.05;

*** *P* < 0.001

Table 2 summarized the correlation between clinical characteristics and radiotherapy of SCEC patients. These characteristics included sex, race, stage, year, age, and surgery. All the characteristics except for disease stage were independent of radiotherapy. Radiotherapy was more likely to be used in the lower disease stage (*P* < 0.001).

**Table 2:** Independence analysis between radiotherapy and other characteristics in SCEC

Variable		NRT	RT	χ^2^	*P*-value^ψ^
Sex	Male	98 (46.67%)	112 (53.33%)	1.8232	0.1769
	Female	77 (54.61%)	64 (45.39%)		
Race	Black	32 (49.23%)	33 (50.77%)	1.3394	0.5118
	White	134 (50.57%)	131 (49.43%)		
	Other	7 (36.84%)	12 (63.16%)		
Stage	Localized	22 (34.92%)	41 (65.08%)	16.332	0.0003***
	Regional	20 (34.48%)	38 (65.52%)		
	Distant	102 (58.62%)	72 (41.38%)		
Year	1973-1992	42 (24.00%)	50 (28.41%)	0.883	0.643
	1993-2002	52 (29.71%)	49 (27.84%)		
	2003-2012	81 (46.29%)	77 (43.75%)		
Age	70−	88 (45.60%)	105 (54.40%)	2.7476	0.0974
	70+	87 (55.06%)	71 (44.94%)		
Surgery	None	156 (50.32%)	154 (49.68%)	0.0000	1.0000
	Surgery	15 (51.72%)	14 (48.28%)		

Abbreviations: MS = median survival; NRT = None radiation therapy; RT = Radiotherapy.

ψ chi-square test;

*** *P* < 0.001

**Figure 1:**
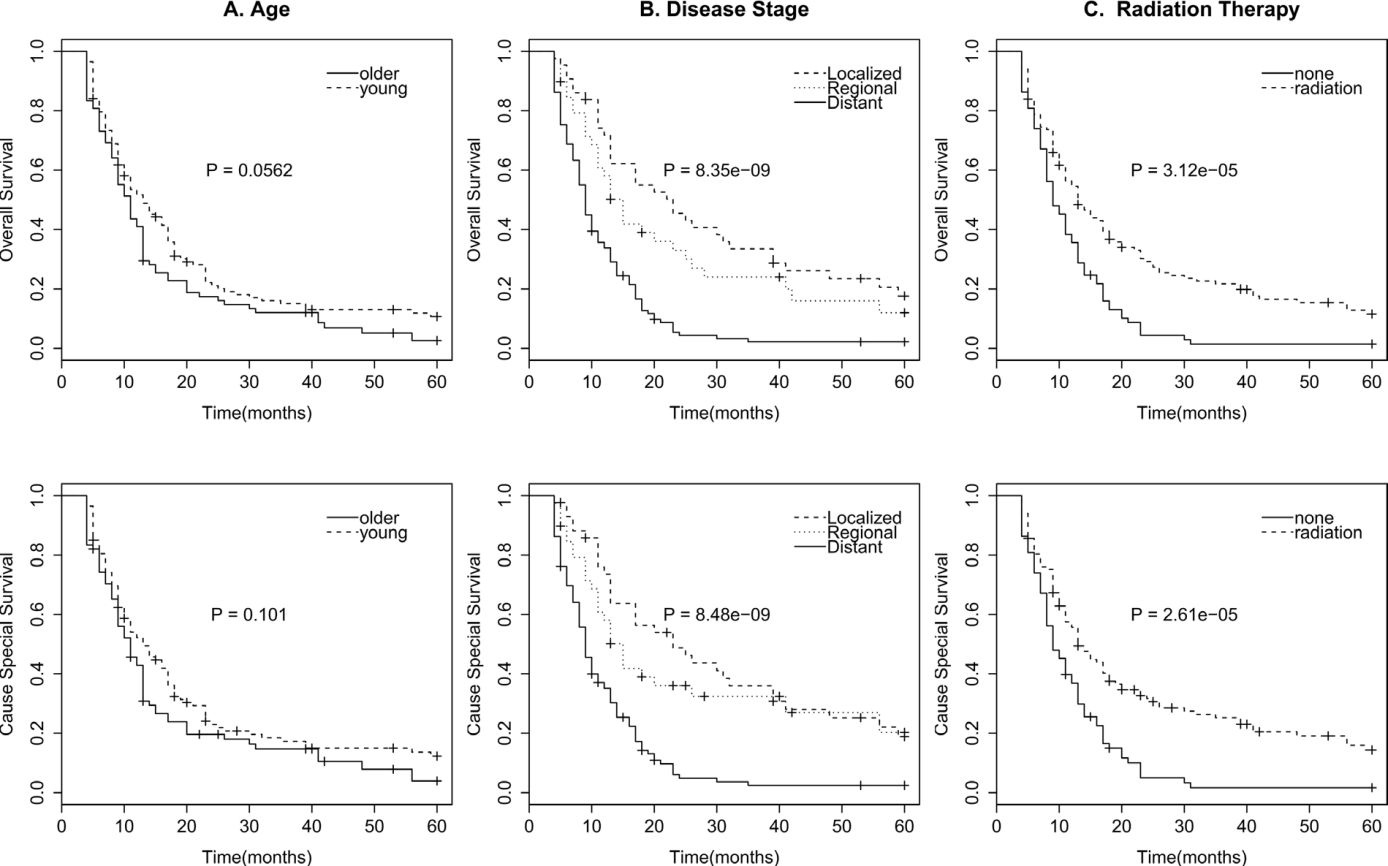
Survival curves in patients according to age (A), Disease stage (B), and radiation therapy (C) of OS and CSS

For the survival analysis of SECE, we excluded patients lived fewer than 4 months to get patients who survived long enough to receive cancer-directed therapy. As surgery and radiotherapy are both local-regional therapy, surgery may be an interference factor when analyzing the survival benefit of radiotherapy. Considering that patients who accepted surgery were very few (only 29 patients), we also excluded patients accepted surgery to eliminate the effect of surgery to radiotherapy. Besides that, unknown stage, race, radiotherapy were also excluded in the survival analysis. Finally, we get 191 patients for survival analysis. Survival effect of clinical characteristics were evaluated with the univariate log-rank test (Table 3). Stage (Figure 1B), and radiation therapy (Figure 1C) were significant associated with OS and CSS (*P* < 0.001). Age (Figure 1A) was possible to be associated with OS (0.05 < *P* < 0.1), but of no association with CSS. Sex, race, and year showed no significant association with survival (*P* > 0.1).

**Table 3:** Univariate survival analyses of SCEC patients

Variable	CSS		OS	
	χ^2^	P-value^†^	χ^2^	P-value^†^
Sex	0.1	0.774	0	0.915
Race	0.7	0.700	0.4	0.830
Stage	37.2	0.000***	37.2	0.000***
Year	0.9	0.624	1.3	0.531
Age	2.7	0.101	3.6	0.056^‡^
Radiation	17.7	0.000***	17.3	0.000***

Abbreviations: MS = median survival; CI = confidence interval.

† Log–rank test.

‡ 0.05 < *p* < 0.1;

*** *P* < 0.001

After that, Multivariate analysis was performed by the Cox regression model (Table 4). The results showed that stage, year, age, and radiation therapy were all independent prognostic factors of OS and CSS (*P* < 0.05), while sex and race were not (*P* > 0.05). Patients younger than 70 years, with lower degree of disease stage, being treated in a later year, and accepted radiotherapy were believed to have a longer cause special survival and overall survival.

**Table 4:** Multivariate cox proportional hazards regression analysis of SCEC patients

Variable	CSS		OS	
	HR (95% CI)	P-value	HR (95% CI)	*P*-value
**Sex**				
Male Vs Female	1.331 (0.936–1.893)	0.111	1.264 (0.897–1.781)	0.180
**Race**				
White Vs Black	0.697 (0.454–1.070)	0.099	0.734 (0.482–1.116)	0.148
Other Vs Black	1.044 (0.524–2.079)	0.904	1.025 (0.517–2.032)	0.945
**Stage**				
Localized Vs Distant	0.258 (0.162–0.411)	< 0.001***	0.252 (0.160–0.396)	< 0.001***
Regional Vs Distant	0.415 (0.263–0.657)	< 0.001***	0.442 (0.286–0.683)	< 0.001***
**Year**				
1993-2002 Vs 1973-1992	0.612 (0.381–0.982)	0.042*	0.622 (0.394–0.982)	0.041*
2003-2012 Vs 1973-1992	0.513 (0.323–0.815)	0.005***	0.509 (0.325–0.798)	0.003***
**Age**				
70− Vs 70+	0.497 (0.346–0.714)	< 0.001***	0.492 (0.345–0.701)	< 0.001***
**Radiation**				
Radiation Vs None	0.547 (0.385–0.778)	< 0.001***	0.559(0.396–0.789)	< 0.001***

Abbreviations: HR = hazard ratio; CI = confidence interval.

* *P* < 0.05;

** *P* < 0.01;

*** *P* < 0.001

**Figure 2:**
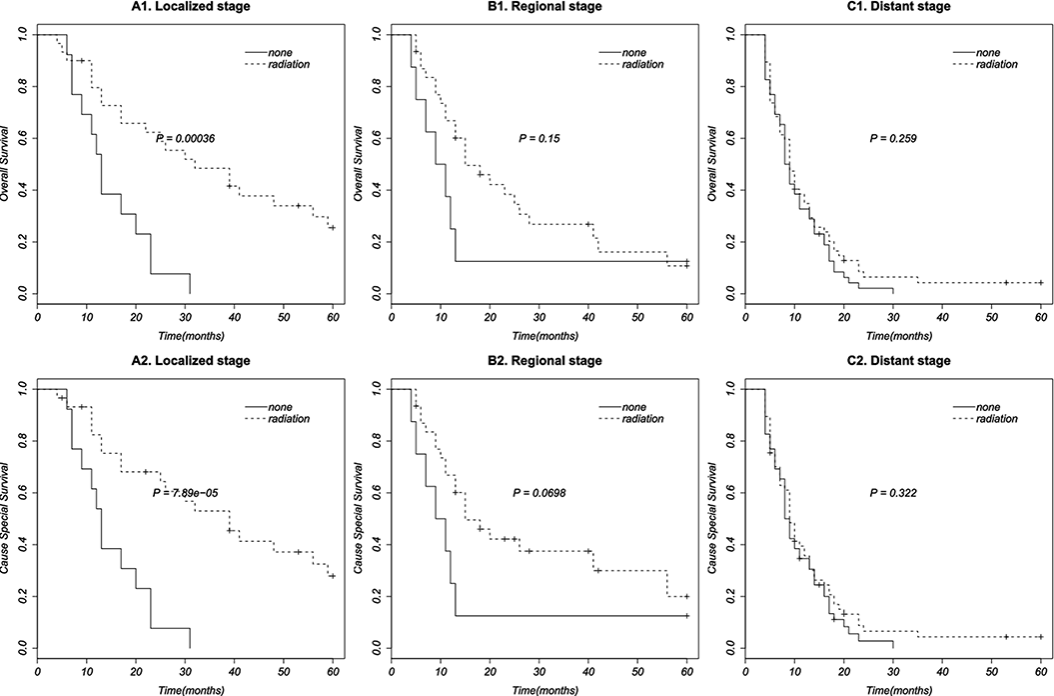
Survival curves in patients according to radiation therapy based on different disease stages

Finally, we perform further multivariate cox regression analysis to assess the efficacy of radiation therapy to OS and CSS based on different stages, by adjusting for sex, race, year, and age (Table 5). The results displayed that radiation therapy can significantly improve OS and CSS in localized stage of SCEC (HR 0.227, 95% CI 0.091–0.566) (HR 0.279, 95% CI 0.117–0.668), but cannot significantly improve OS and CSS in regional (HR 0.413, 95% CI 0.153–1.118) (HR 0.473, 95% CI 0.180–1.241) and distant stages (HR 0.700, 95% CI 0.458–1.068) (HR 0.683, 95% CI 0.4498–1.037). Survival curves of radiation therapy based on different disease stages were established by Kaplan-Meier method and showed in Figure 2.

**Table 5:** Multivariate cox proportional hazards regression analysis of radiotherapy based on different stages of SCEC

Stage	CSS		OS	
	HR (95% CI)	*P*-value	HR (95% CI)	*P*-value
**Localized**				
Radiation Vs None	0.227 (0.091–0.566)	0.001**	0.279 (0.117–0.668)	0.004**
**Regional**				
Radiation Vs None	0.413 (0.153–1.118)	0.082	0.473 (0.180–1.241)	0.128
**Distant**				
Radiation Vs None	0.700 (0.458–1.068)	0.098	0.683 (0.4498–1.037)	0.073

Abbreviations: HR = hazard ratio; CI = confidence interval.

** *P* < 0.01

## DISCUSSION

Small cell esophageal carcinoma is rare, aggressive and poor prognostic. It is reported to be only 0.5%-2.1% of all esophageal cancers [3, 10–12]. This results in few large sample clinical studies of SCEC. Currently, clinical treatment strategies of SCEC are very limited and full of contradiction [3, 5, 6, 13]. As SCC is always regarded as a systemic disease [1], chemotherapy is a unique systemic therapy of multimodality treatment of SCEC [1, 6, 7, 14–16]. Thus, main dispute of treatment of SCEC is in the locoregional therapy. Many studies [8, 17, 18] recommend chemotherapy and surgery as primary treatment of limited disease, while others [9, 19] suggested chemoradiotherapy was better, also someone [3, 7] discovered similar effect of chemotherapy with radiotherapy or surgery. To our knowledge, so far, there is still not a clear evaluation of radiotherapy to SCEC, neither in limited stage, nor in extensive stage.

In this study, we summarized the clinical characteristics of SCEC with information provided by the population-based SEER database from 1973 to 2012. The characteristics used for analysis in our study contained sex, race, disease stage, year, age, radiation therapy, and surgery. We found that SCEC was rare (0.58%) in esophagus tumors. It tended to be higher stage, older, female and have a shorter survival in compare with NSCEC, which is in conformity with the previous literature [3]. More than half of SCEC patients were treated with radiation therapy, but only fewer than 10% of whom were in surgery. Low surgery proportion made it improper to analyze the effect of surgery, so we only analyzed the survival benefit of radiotherapy in this study by excluding the patients performed surgery. Independence chi-square test between radiation therapy and other factors showed that radiation therapy was associated with disease stage. Radiation therapy was more likely to be used in localized (65.08%) and regional (65.52%) stages than in distant stage (58.62%). Univariate survival analyses showed that OS was associated with stage, radiotherapy (*P* < 0.001), and possible age (0.05 < *P* < 0.1), but not associated with sex, race, and year (*P* > 0.1). It is in agreement with Mansoor et al's study [3]. Multivariate cox proportional hazards regression analysis displayed that year, age, disease stage, and radiation therapy were all significantly associated with OS and CSS (*P* < 0.05). As the results of OS and CSS in multivariate analysis were similar, We take OS as an example for the following discussion. Patients had a lower risks of death in localized stage (HR 0.252, 95% CI 0.160–0.396) and regional stage (HR 0.442, 95% CI 0.286–0.683) compared with in distant stage. Death risk of patients younger than 70 years was less than half (HR 0.492, 95% CI 0.345–0.701) of those older than 70. Patients being diagnosed after 2003 (HR 0.509, 95% CI 0.325–0.798) and during 1993 to 2002 (HR 0.622, 95% CI 0.394–0.982) had less death risk of those in 1973 to 1992. It may due to the development of radiotherapy technology. Radiation could reduce nearly 50% of death hazards (HR 0.559, 95% CI 0.396–0.789). Further multivariate analysis of prognostic factors based on different stages showed that radiotherapy can reduce 72,1% risks of death in localized stage (HR 0.279, 95% CI0.117–0.668), 52.7% risks of death in regional stage (HR 0.473, 95% CI 0.180–1.241) and more than 30% risks of death in distant stage (HR 0.683, 95% CI 0.450–1.037).

One should be mentioned is that chemotherapy was not enrolled in the survival analysis. It was due to the lack of the record of chemotherapy in the SEER database. For the results that SCEC is always regarded as a systemic disease and suggested to be treated with multimodality treatment for many years [12, 14, 20, 21], we have reasons to assume that most patients who lived more 3 months had enough time to accept chemotherapy. Thus, in this study, we excluded all patient who lived fewer than 4 months to minimize the effect of chemotherapy to radiotherapy.

In conclusion, our study demonstrates that year, age, disease stage, and radiotherapy are all independent prognosis factors of SCEC. Age and disease stage are negative associated with OS and CSS. Year and radiation therapy are positive associated with OS and CSS. Further analysis based on different disease stages showed that survival benefit of radiotherapy existed in any disease stage, but was only statistically significant in localized stage of SCEC.

## MATERIALS AND METHODS

### Patients

SEER data between 1973 and 2012 [“Incidence - SEER 18 Regs Research Data + Hurricane Katrina Impacted Louisiana Cases, Nov 2014 Sub (1973–2012 varying)”] were chosen for this study. The latest National Cancer Institute's SEER*Stat software (Version 8.2.1) was used for the identity of patients with small cell histology (Histologic/Behavior codes: 8041/3 and 8043/3) and esophageal tumor (Site recode: Esophagus). Survival data were extracted at 1-month intervals for a minimum follow-up of 4 months and a maximal follow-up of 60 months to exclude patients who did not survive long enough to receive cancer-directed therapy. Those who accepted surgery were also excluded for reducing the effect of surgery to radiotherapy.

This study was based on public data from the SEER database. The reference number we obtained for the permission to access research data files was 10612-Nov 2014. No human subjects or personal identifying information were used in this study. No informed consent was require in this study. This study was approved by the Review Board of Huai'an First People's Hospital, huai'an, China.

### Statistical analysis

Chi-square test was used to analyze the difference between SCEC and NSCEC, and the correlation between radiotherapy and other factors. Univariate analyses with log-rank test and multivariate analysis with cox proportional hazards regression model were performed to examine the clinical factors’ association with cause-specific survival (CSS) and overall survival (OS) respectively, with a statistically significant difference at *p* < 0.05. The factors included age, sex, race, stage, and radiation therapy. Finally, cox regression analysis of radiotherapy were performed based on different disease stages. All analysis were performed in the population with a whole record of analytical variable. All analysis were performed with survival package [22, 23] of R [24] (version 3.2.1).
